# Belowground mechanism reveals climate change impacts on invasive clonal plant establishment

**DOI:** 10.1038/s41598-022-06918-w

**Published:** 2022-02-21

**Authors:** Surendra Bam, Jacqueline P. Ott, Jack L. Butler, Lan Xu

**Affiliations:** 1 Department of Ecology, Environment and Evolution, La Trobe University, Melbourne, VIC 3086 Australia; 2 Forest and Grassland Research Laboratory, US Forest Service, Rocky Mountain Research Station, Rapid City, SD USA; 3 Department of Natural Resource Management, South Dakota State University, Brookings, SD USA

**Keywords:** Grassland ecology, Invasive species, Population dynamics

## Abstract

Climate change and disturbance can alter invasion success of clonal plants by differentially affecting the clonal traits influencing their establishment as young plants. Clonal traits related to the vegetative reproduction of native *Pascopyrum smithii* and non-native *Bromus inermis* grass seedlings were evaluated under altered precipitation frequencies and a single grazing event. *Pascopyrum smithii* maintained similar vegetative reproduction under three simulated precipitation frequencies whereas *B. inermis* vegetative reproduction declined as precipitation became more intermittent. Vegetative reproduction of the non-native *B. inermis* was greater than the native *P. smithii* under all simulated precipitation frequencies except the most intermittent scenario. A single grazing event did not affect either species’ response to intra-annual precipitation variability but did slightly reduce their clonal growth and increase their bud dormancy. In young plants, clonal traits of the invasive grass favored its superior expansion and population growth compared to the native grass except under the most severe climate change scenario. Grassland restoration using native *P. smithii* seeds would be successful in most years due to its resilient clonal growth in a changing climate. Clonal infrastructure development in young plants is critical to clonal plant establishment and persistence in a changing climate and under disturbed conditions.

## Introduction

Biotic invasions can have far-reaching and long-term consequences on ecosystem function and biodiversity^1–3^. For a non-native species to be successful in its introduced range, it must survive multiple hurdles: from dispersal to colonization to establishment to landscape spread^4, 5^. Although numerous abiotic and biotic filters determine which species can colonize a system, species-specific biological traits often determine their local persistence and rate of spread following introduction^4, 6^.

Clonal growth, a morphological and demographic characteristic of many invasive plant species, enables rapid colonization of new locations through physiological integration among ramets to ensure successful vegetative expansion^7–9^. Non-native clonal species often invade into established native systems such as perennial grasslands, where the dominant native species are also clonal. Clonal species are a specialized group of perennial species which can produce self-sufficient offspring vegetatively^10^. Therefore, the relative differences in invaders' clonal characteristics compared to their native counterparts may be nuanced, with invader success dependent on specific differences such as ramet longevity, the persistence of clonal connections, degree of physiological integration, bud bank size, or carbohydrate supply in the clonal growth organ^8, 11^. Resource sharing facilitated by clonal integration has been shown to increase the performance of invasive species more than native species^12^. Propagule pressure during invasion is often measured in terms of seed production but rarely encompasses vegetative reproductive propagules, such as buds and plant fragments, produced and used by clonal plant populations to colonize and spread in new habitats^13–15^. Invasive clonal species may invest more into “clonal infrastructure” than native species through increased bud and ramet production, especially at the beginning of plant establishment^14^. Flexible timing of bud and ramet production by generating multiple generations within a single year would enable a species to be responsive to changing resource conditions and to not rely on a single ramet recruitment and bud production time period.

In clonal species, plant architectural development of seedlings through vegetative reproduction (i.e., clonal growth via the addition of new ramets) is key to continued growth and expansion^16^. Well-established adult clonal plants benefit from extensive physiological integration among ramets^17^. Young clonal plants need to create this extensive integration, which, in the case of invasive species, can be used to facilitate their invasion^18^. Plants are most sensitive to their environments at early stages of their life history^19^. Successful plant establishment and persistence depends on resource availability paired with the plant structural development needed to acquire these resources.

Extreme weather events resulting from climate change can provide windows of opportunity to enhance biological invasion by decreasing the resistance of native communities to invasive species establishment and promoting the reproduction of invasive species^20–22^. The fluctuating resource hypothesis suggests that invasion success is positively related to resource pulses, such as increased intra-annual variability in precipitation^23–25^. Understanding how extreme weather events or more variable climatic conditions impact the relative performance of native and non-native species, especially at early life history stages, could offer insight into the potential success of invasive clonal plant species under future climate change scenarios.

Invasive species can be difficult to control when they have similar photosynthetic pathways and growth forms as the dominant native species of the invaded community^26^. For example, the invasive C_3_ (i.e. cool-season) grasses with their earlier growing season can be controlled using spring fires when in a grassland dominated by native C_4_ (i.e. warm-season) that have a later summer growing season^27^. When C_3_ invasives invade C_3_ dominated grasslands, spring fire and other grazing treatments will similarly hinder both the native and invasive species^26^. *Bromus inermis* Leyss. and *Pascopyrum smithii* (Rydb.) Á. Löve offer a direct comparison of a native and an invasive species. The invasion of *B. inermis* into native *P. smithii* stands in the Northern Mixed-Grass Prairie is a growing concern as *B. inermis* invasion rapidly displaces the dominant native species and homogenizes the plant community and modifies the soil^28–30^ causing loss of species diversity and reduced habitat use by native ungulates^31, 32^. The success of *B. inermis* may be due to subtle but important differences in clonal traits, such as multiplication rate and bud bank size^11^, which could vary with grazing pressure and variability in intra-annual precipitation.

Grassland ecosystems, with their major drivers of precipitation and grazing, are excellent systems to compare invasive and native seedling development under increased climatic variability and disturbance^33^. Moderate inter-annual variability in precipitation pushes these systems between periods of water surplus and water scarcity^34^. The predicted increase in intra-annual precipitation variability in North American grasslands will produce larger individual precipitation events with longer intervening dry periods^35^. Such changes will generate more temporally dynamic soil moisture regimes creating more stressful conditions for native plants and opportunities for invasion^25, 36^. Clonal grass buds often occur belowground, protecting them from direct mortality by aboveground grazers^37^. Still, aboveground defoliation can affect belowground bud dormancy and recruitment of new tillers from the bud bank^14^. Clonal grasses can cope with herbivory and precipitation variability by shifting periods of tiller recruitment and altering placement of meristems and axillary buds^37–41^.

In order to examine the potential effects of climate change and disturbance on vegetative reproduction and propagule production of invasive as compared to native seedlings, the effects of precipitation frequency (to simulate intra-annual precipitation variability) and grazing on vegetative reproduction and expansion of *B. inermis* and *P. smithii* seedlings was examined during their first growing season using a temperature-controlled greenhouse. Specifically, this study examined two questions:

1. How does the vegetative reproductive development in seedlings of the dominant native C_3_ clonal grass *P. smithii* compare to the invasive clonal C_3_ grass *B. inermis* in ungrazed conditions when receiving annual precipitation through frequent, small precipitation events?

Similar to adult plants^42^, we hypothesized that the invasive *B. inermis* will have greater vegetative reproductive development than the native *P. smithii* through increased tiller and rhizome production and propagule production during its first growing season. Rapid clonal development of seedlings would enable the invasive species to establish and expand in a new location.

2. Does increasing intra-annual precipitation variability, grazing, or their combination alter the vegetative reproductive development of these two species?

Based on the fluctuating resource hypothesis, invasive species benefit from resource pulses. We hypothesized that invasive *B. inermis* seedlings will increase vegetative reproductive development more than native *P. smithii* seedlings as intra-annual precipitation variability increases. We further hypothesized that grazing of young seedlings will decrease vegetative reproductive development similarly in both species but more under high intra-annual precipitation variability. Loss of photosynthetic tissue will reduce carbon fixation needed for clonal growth and recovery of this tissue would be hampered by variable water resources.

## Results

### Plant establishment through clonal growth

**Figure 1:**
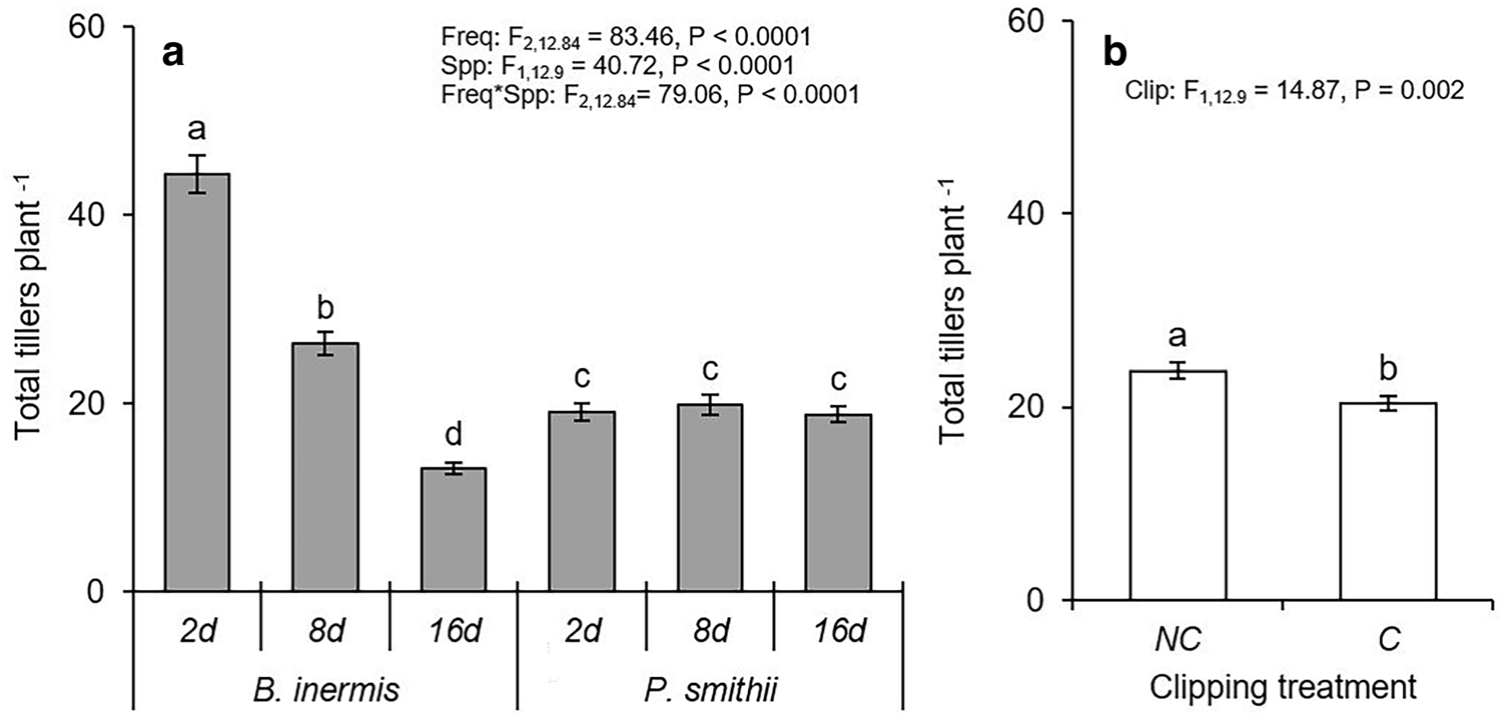
Effect of (**a**) precipitation frequency and (**b**) clipping on the number of total tillers per plant of *Bromus inermis* and *Pascopyrum smithii*. Values are mean ± SE based upon the statistical model. Significant differences at *p*-value < 0.05 indicated by different letters. Interaction results are shown when *p*-value < 0.05. Full results are shown in Table S1.

Plants of both species survived the entire experiment, but grew differently depending on precipitation frequency. Precipitation frequency did not affect tiller production of the native species *P. smithii*, but did reduce tiller production of the invasive *B. inermis* as time between simulated precipitation events increased (Fig. 1a). However, as long as watering occurred regularly (every 2 or 8 days), the invasive species produced more tillers than the native species (Fig. 1a). When watering was infrequent (every 16 days), the native *P. smithii* outperformed the invasive species producing approximately five more tillers per plant. The single clipping event significantly reduced tiller production similarly for each species regardless of precipitation frequency (Fig. 1b).

**Figure 2:**
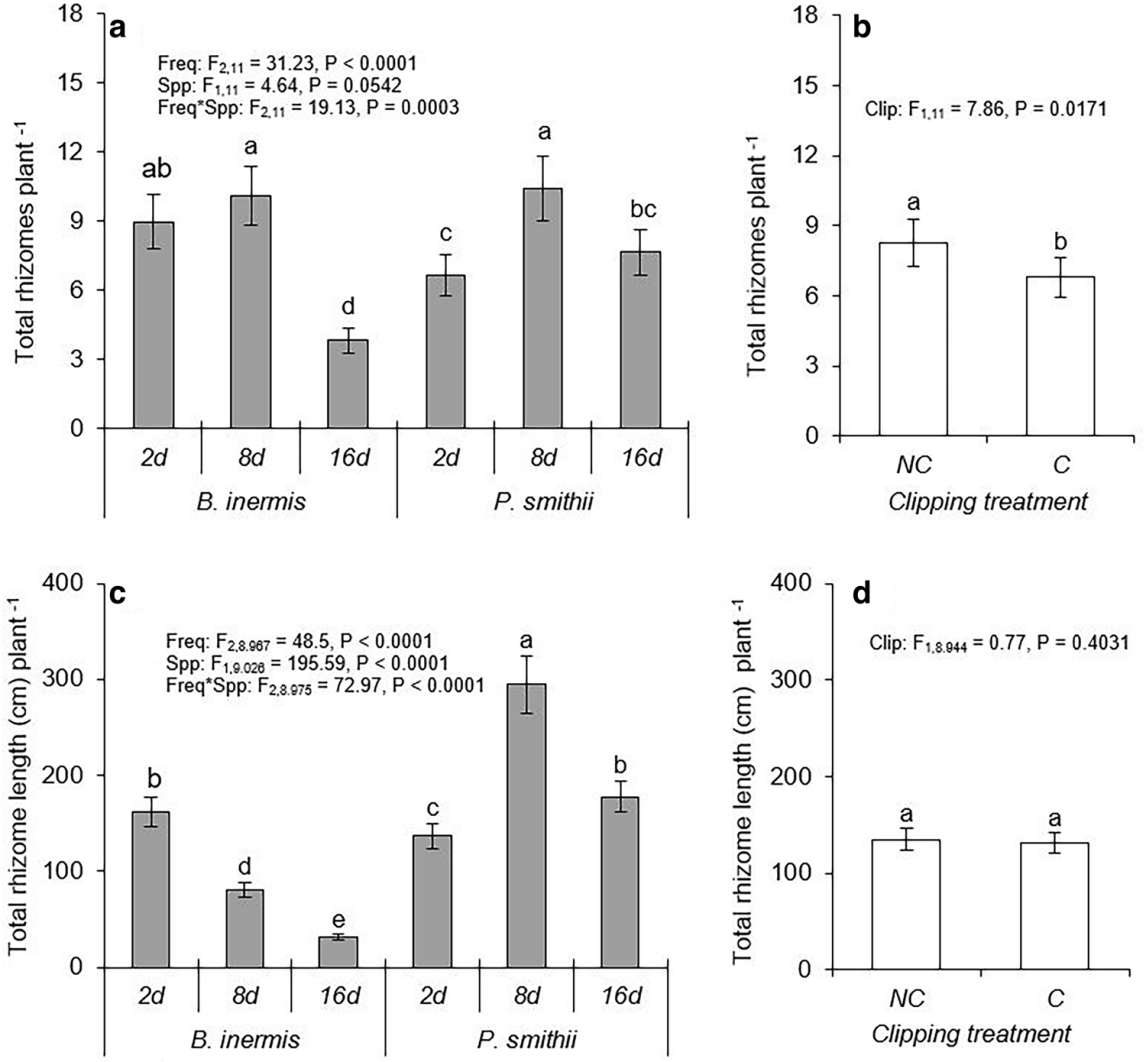
Effect of precipitation frequency and clipping on number of total rhizomes (**a**, **b**) and total rhizome length (**c**, **d**) per plant of *Bromus inermis* and *Pascopyrum smithii*. Values are mean ± SE based upon the statistical model. Significant differences at *p*-value < 0.05 indicated by different letters. Interaction results are shown when *p*-value < 0.05. Full results are shown in Table S2 and S3.

Precipitation frequency affected rhizome demography of *B. inermis* and *P. smithii* differently. At moderate and high precipitation frequencies, *B. inermis* produced similar rhizome numbers, but rhizome numbers were reduced by 50% under the lowest precipitation frequency (Fig. 2a). The native *P. smithii* had its greatest number of rhizomes at the intermediate watering frequency (Fig. 2a). At the lowest precipitation frequency, *P. smithii* produced more rhizomes than *B. inermis*. Patterns in rhizome length mirrored patterns in rhizome demography for *P. smithii* (Fig. 2c). However, *B. inermis* plants steadily reduced their total rhizome length as watering became less frequent. Unlike the native *P. smithii*, *B. inermis* initially reduced rhizome length rather than rhizome number as watering became more infrequent. Clipping lowered rhizome production similarly in both species across all precipitation frequencies (Fig. 2b) but did not affect rhizome length (Fig. 2d).

### Tiller replacement

**Figure 3:**
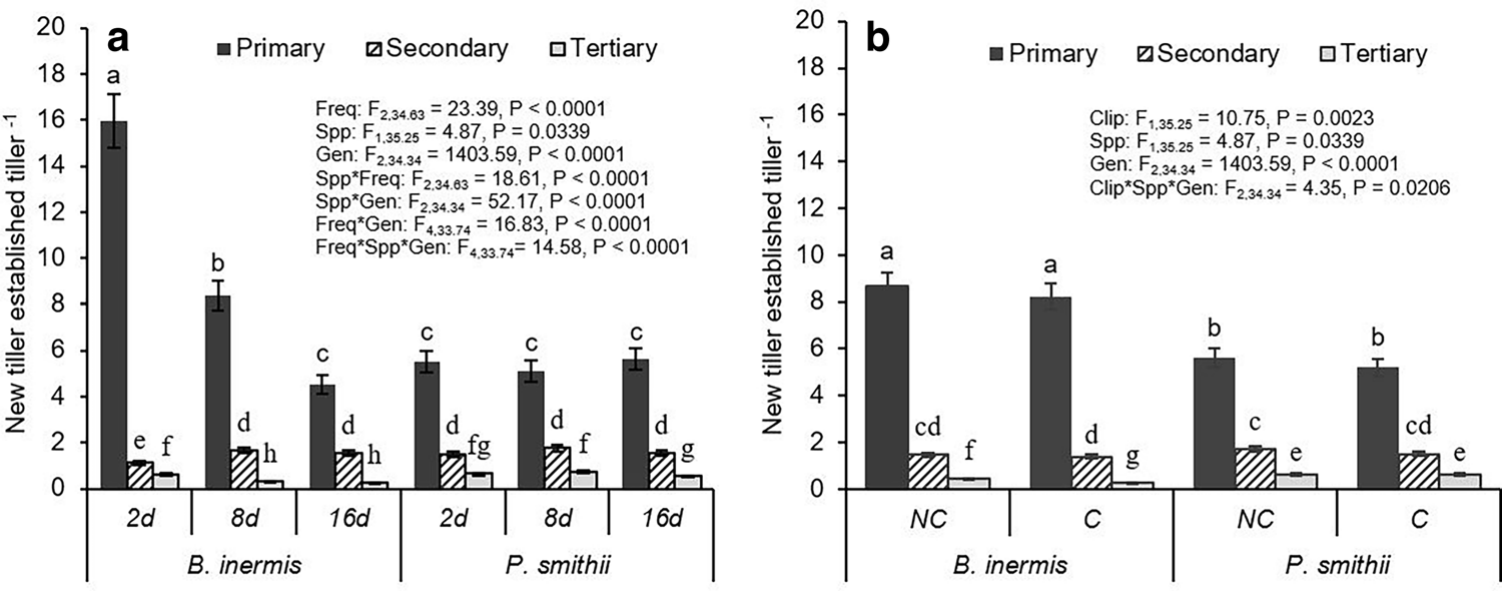
Effect of (**a**) precipitation frequency and (**b**) clipping on number of new tillers established per tiller of *Bromus inermis* and *Pascopyrum smithii*. We had three daughter tiller generation cohorts defined as primary, secondary, and tertiary tiller generation (see Fig. 1 for conceptual diagram). Significant differences at *p*-value < 0.05 indicated by different letters. Interaction results are shown when *p*-value < 0.05. Full results are shown in Table S4.

Each species produced three generations of tillers during their first growing season. Seedlings of both species produced between four to sixteen primary generation tillers whereas subsequent generations (secondary and tertiary) produced far fewer tillers per parent tiller (e.g., one secondary tiller per primary tiller or one tertiary tiller per secondary tiller; Fig. 3a). Secondary tiller production was always greater than one (i.e., capable of sustaining a secondary tiller population size similar to its parent primary tiller generation), but tertiary tiller production was always less than one. As precipitation frequency decreased, *B. inermis* seedlings produced significantly fewer primary tillers, while primary tiller production of *P. smithii* seedlings was unaffected by precipitation frequency (Fig. 3a). Clipping had little effect on primary and secondary tiller production of either species, and on tertiary tiller production of *P. smithii*. Clipped *B. inermis* produced significantly fewer tertiary tillers compared to unclipped *B. inermis* (Fig. 3b).

### Propagule supply and development

**Figure 4:**
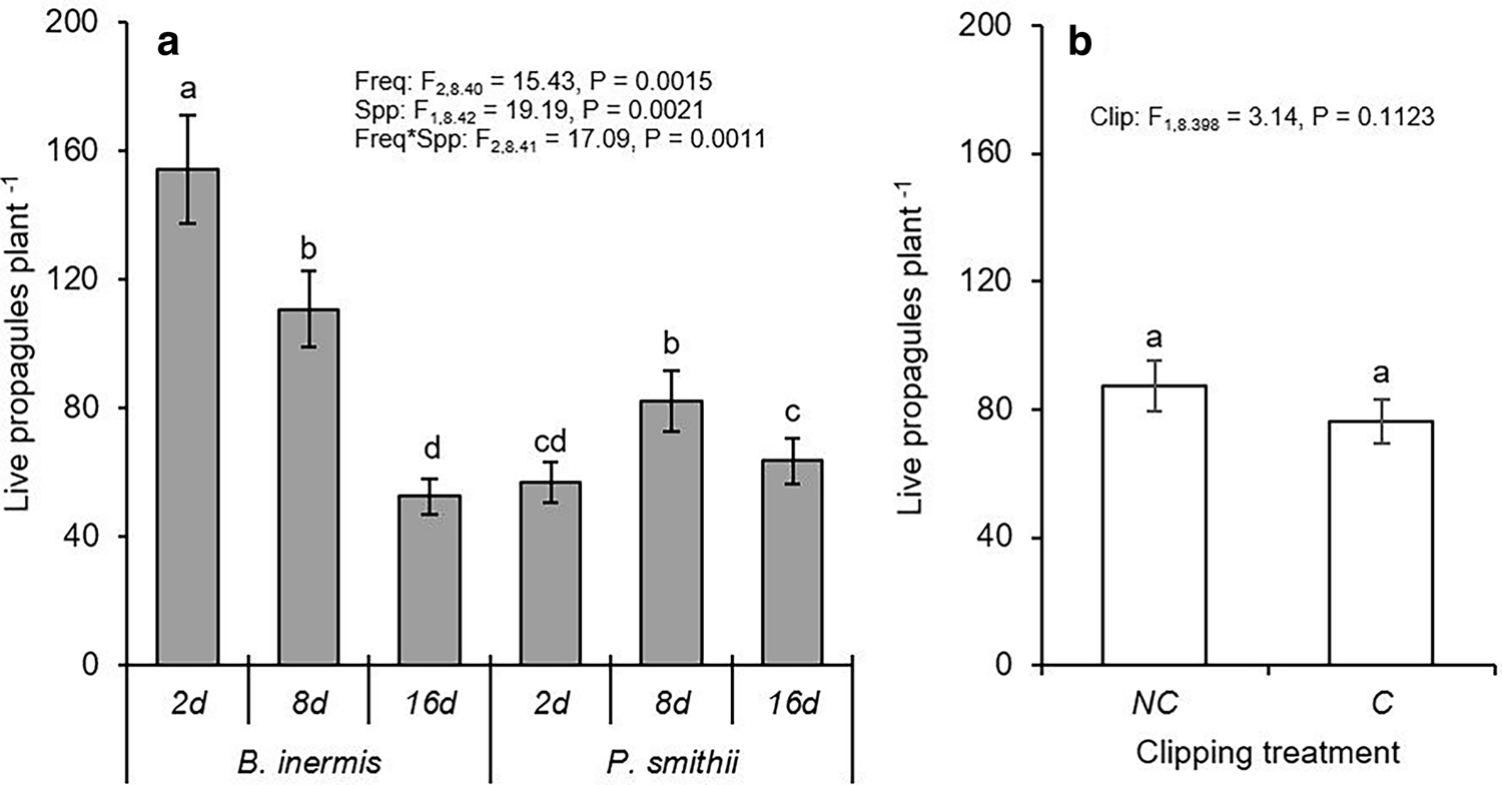
Effect of (**a**) precipitation frequency and (**b**) clipping on number of live propagules per plant of *Bromus inermis* and *Pascopyrum smithii.* Values are mean ± SE based upon the statistical model. Significant differences at *p*-value < 0.05 indicated by different letters. Interaction results are shown when *p*-value < 0.05. Full results are shown in Table S5.

**Figure 5:**
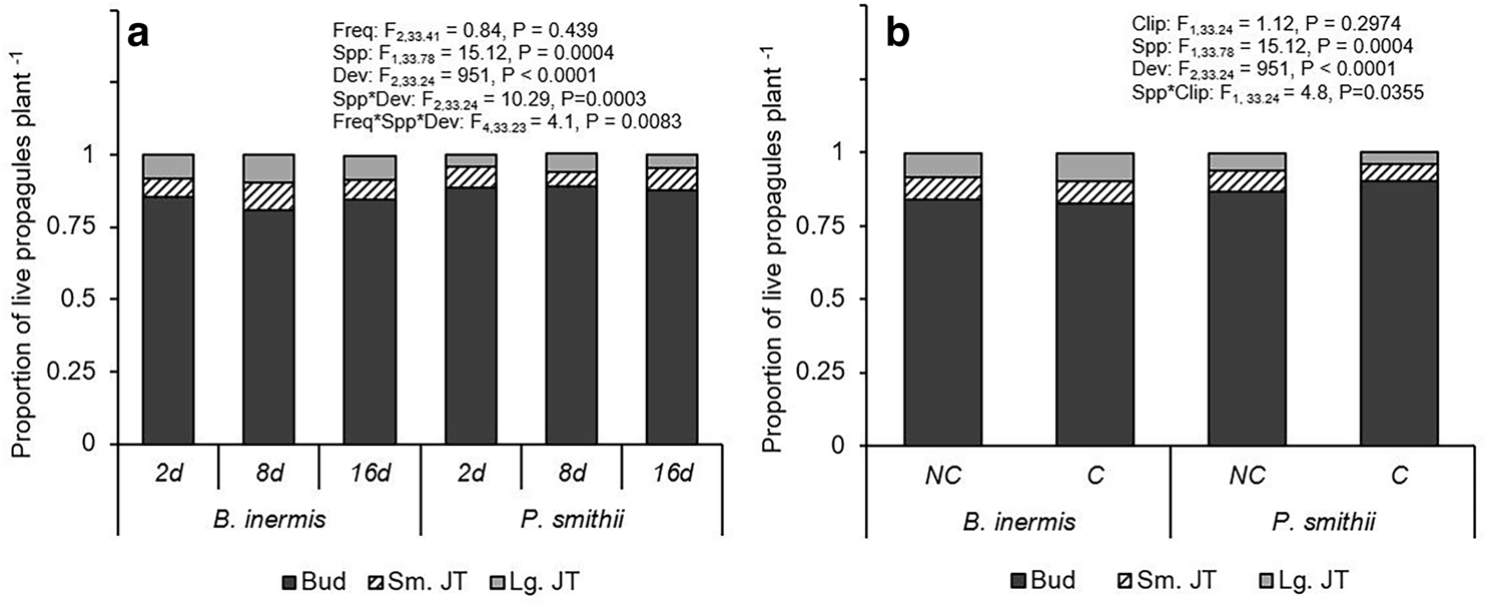
Effect of (**a**) precipitation frequency and (**b**) clipping on proportion of live propagules at each developmental stage per plant of *Bromus inermis* and *Pascopyrum smithii*. The proportions of live propagules were classified into three development/size classes including buds, small juvenile tillers/rhizomes (Sm. JT), and large juvenile tillers/rhizomes (Lg. JT). Interaction results are shown when *p*-value < 0.05. Full results are shown in Table S6.

The invasive *B. inermis* produced more propagules than the native *P. smithii* at the highest precipitation frequency (Fig. 4a). Otherwise *P. smithii* maintained similar or more propagules per plant than invasive *B. inermis* at moderate and low precipitation frequencies. Propagule development in *B. inermis* was significantly greater than *P. smithii* at all watering frequencies (Fig. 5a). Across precipitation frequencies, *B. inermis* transitioned 3 to 9% more buds to juvenile tillers than *P. smithii*. *Pascopyrum smithii* tended to maintain a consistent percentage of juvenile tillers but whether plants maintained them as small or large juvenile tillers varied (e.g., more large juvenile tillers occurred at the intermediate precipitation frequency). *Bromus inermis* maintained a consistent percentage of large juvenile tillers across precipitation frequencies and transitioned a greater percentage of buds to small juvenile tillers at the intermediate precipitation frequency (Fig. 5a). Although clipping did not affect the size of the propagule supply for either species (Fig. 4b), clipping significantly reduced propagule development in *P. smithii* with 4% of propagules remaining as dormant buds rather than transitioning into juvenile tillers (Fig. 5b).

## Discussion

### Clonal plant establishment and invasion under climate change

In support of our hypothesis, invasive *B. inermis* seedlings had greater vegetative reproductive development than native *P. smithii* seedlings under ungrazed conditions with frequent, small precipitation events. Under these conditions, *B. inermis* displayed exponential tiller population growth, greater rhizome production, and longer rhizomes than the native *P. smithii*. In contradiction to our second hypothesis and the fluctuating resource hypothesis, clonal growth of invasive *B. inermis* seedlings declined with increasing intra-annual precipitation variability and aligns with the decreased establishment and seedling persistence observed in a field study of *B. inermis*^25^. Based on our results, successful clonal growth following seedling establishment may be limited unless precipitation is evenly distributed throughout the growing season. Consequently, increased intra-annual precipitation variability predicted by climate change models for the Great Plains would hinder *B. inermis* expansion from their colonization sites. In contrast, *P. smithii* maintained steady clonal growth across the three precipitation frequencies. The moderate inter- and intra-annual variability in precipitation of Great Plains grassland systems^34, 43^ has likely conditioned and favored native species that maintain consistent clonal growth of seedlings. Restorations using *P. smithii* seeding in mixed-grass prairie should be successful in most years as long as average precipitation is not greatly reduced.

The spatial expansion of young invasive seedlings via rhizome growth declined with increasing intra-annual precipitation variability. Native seedling rhizome expansion peaked at an intermediate precipitation frequency indicating each species had different expansion strategies. Native seedlings exhibited an efficient strategy, only increasing expansion when the plant benefits from wider spatial occupancy but not overextending in dry conditions. Invasive seedling rhizome expansion was risk-averse, only expanding when water availability was stable but maintaining architectural plasticity by initially sacrificing rhizome length rather than rhizome number as time between precipitation events increased. Seedlings may show greater investment in expansion than adult plants. Young *P. smithii* seedlings produced more tiller generations and approximately 7 × the number of rhizomes in this study as adult *P. smithii* plants^44^. Rhizome demography can also be affected by differences in growing conditions^45, 46^ or in the varieties of each species^47–49^.

Early season clipping had a small but significant reduction in tiller and rhizome production of both species supporting our hypothesis, but did not alter their response to intra-annual precipitation variability failing to support our hypothesis. The statistically significant reduction in tertiary *B. inermis* tillers due to clipping may be biologically insignificant and disappear if plants were allowed to continue growth. Repeated defoliation may negatively affect seedling establishment, as aboveground productivity and relative growth rates often decline with increasing defoliation frequency within the growing season and in consecutive years^50^. Repeated grazing initially increases *P. smithii* tiller recruitment^51^, but usually results in reduced tiller densities by the end of the season^52^. Although not seen in this study, clipping may shift biomass allocation belowground in *B. inermis*^47, 53, 54^.

### Multiple tiller generations during plant establishment

**Figure 6:**
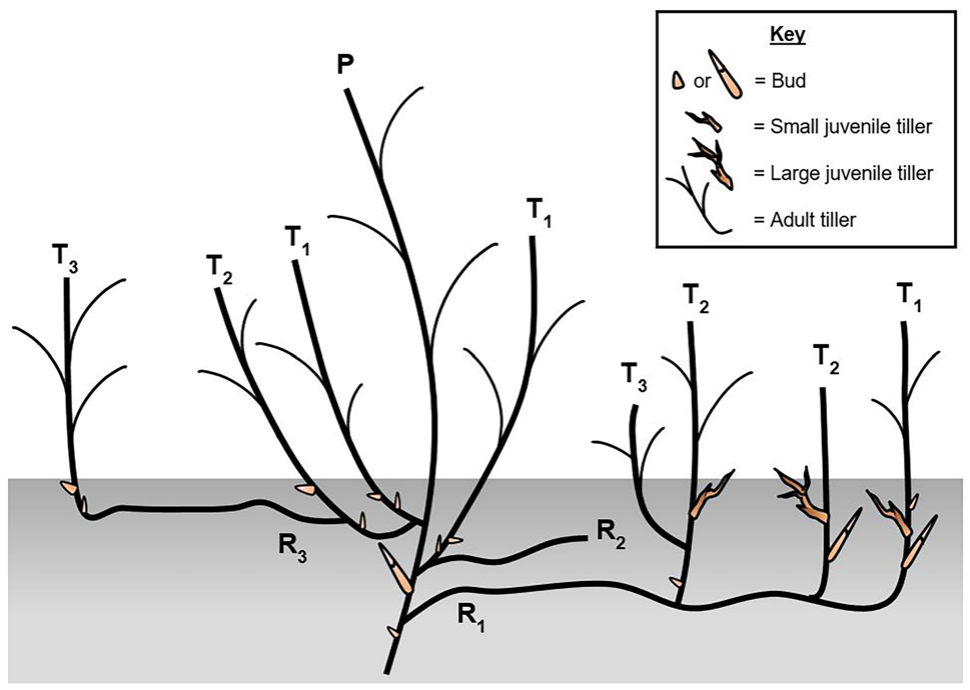
Conceptual diagram of live propagules and tiller and rhizome classification according to generations. R = rhizome, T = adult tiller, P = parent, 1 = primary generation, 2 = secondary generation, 3 = tertiary generation. The parent tiller produced primary rhizomes or tillers which in turn produced secondary rhizomes or tillers.

Both *P. smithii* and *B. inermis* had greater multiplication rates for primary tillers rather than secondary or tertiary tiller generations, underscoring the importance of initial vegetative reproduction of ramets to plant establishment. Although secondary and tertiary tiller production was steady, secondary tiller production occurred at rates adequate to replace the first generation of tillers and maintain its population size rather than grow its population size. Tertiary tiller production yielded a smaller tiller population size than previous generations. Multiple stem generations are likely a characteristic of young plants as established plants of both species typically produce one annual generation of tillers^44^. Therefore, reduced bud dormancy in young plants likely enable them to have high tiller recruitment from parent tillers and accelerated recruitment from primary and secondary tiller generations (Fig. 6).

The exponentially greater tiller production of *B. inermis* over *P. smithii* strongly relied on the greater primary tiller productivity (i.e., multiplication rate) of *B. inermis*. Without the high primary tiller generation multiplication rate of invasive *B. inermis*, native *P. smithii* would have similar or greater aboveground clonal growth than *B. inermis*. Young *B. inermis* plants likely exhibited high primary tiller emergence under high water availability due to their nascent developmental stage rather than as a response to early-season grazing or photoperiod (see tillering responses^55, 56^). This large initial investment in tiller production under wet conditions could explain how *B. inermis* is able to rapidly occupy and expand from colonization sites in the field.

### Formation of clonal propagule supply

The *B. inermis* propagule bank size increased as precipitation frequency increased while *P. smithii* propagule bank size was fairly consistent across all precipitation regimes. Clipping did not affect the propagule bank of either species which failed to support our hypothesis. *Bromus inermis*, however, had a larger propagule supply than *P. smithii* only under frequent precipitation, which partially supported our hypotheses. The propagule bank size depends on the number of propagules produced per tiller as well as the number of tillers. Bud production per tiller of *B. inermis* was 2 × greater than *P. smithii* in adult plants^42^. In this study of seedlings, greater bud production per tiller in addition to a greater number of tillers and rhizomes contributed to the larger propagule bank of *B. inermis* under frequent precipitation conditions. Axillary bud production is closely associated with tiller growth, and perennial grass species often have consistent bud production per tiller^14, 57^. This explains why propagule production per plant closely mirrored aboveground tiller and rhizome production responses to precipitation frequency and clipping in this study.

Each species maintained a large percentage (~80%) of its propagule supply as dormant buds. Entering the winter dormant season, these young seedlings released fewer buds into juvenile tiller stages reserving more buds for spring outgrowth. In contrast to these young seedlings, adult plants maintained a relatively smaller percentage of its springtime propagule supply as dormant buds (~ 60%^40^). Cool-season grasses often maintain multiple propagule developmental stages to respond quickly to favorable growing conditions and grow incrementally as conditions allow^14, 57^. Young plants overwintered smaller numbers of belowground juvenile tillers than adult plants, as mortality risk of juvenile tillers may be greater than that of dormant buds. Greater propagule dormancy may also be an artifact of increased tiller recruitment and production of multiple tiller generations as juvenile tillers rapidly transition into adult tillers in these young plants. Moderate precipitation frequency slightly reduced *B. inermis* propagule development but no major shifts were observed in the propagule development of either species. Bud developmental activity may be less responsive to intra-annual precipitation variability than it is to longer, multi-year droughts^58^.

Clipping increased bud dormancy only in the native *P. smithii* seedlings similar to effects observed in adult *P. smithii* plants^42^. Herbivory-induced bud dormancy release may enable short-term compensation but longer-term plant growth will decline with repeated grazing events^59^. Therefore, *P. smithii* may be offsetting the negative effects of herbivory by increasing dormancy to limit tiller recruitment immediately following herbivory and conserve its buds for future outgrowth opportunities.

## Conclusions

Clonal traits, such as lateral vegetative spread, bud bank size, and ramet multiplication rate, are one of the primary factors associated with successful plant invasions^16^. Although species have similar photosynthetic pathways, growth forms, and environmental tolerances, their clonality traits can differ enabling invasive species to outcompete their native counterparts. In this study, young invasive plants had greater clonal growth than the native species under current climate conditions. Climate change can shift plant invasion success by altering the expression of clonal traits of invasive and native species. This experiment found that invasive clonal growth declined under climate change scenarios although native clonal growth remained steady. This research offers hope that grassland restoration using the native *P. smithii* would be successful due to its resiliency in a changing climate. To better understand the mechanisms driving the invasion success of clonal plant species, our focus needs to include clonal traits and their expression in young plants as well as their response to climate change and disturbance. A solitary grazing disturbance in the experiment did slightly reduce clonal growth and increase bud dormancy. The importance of developing clonal infrastructure was highlighted in this study as young plants of both species formed multiple tiller generations and conserved buds through high dormancy rates for future outgrowth opportunities. Clonal traits are necessary to understand invasion patterns and success and need to be incorporated into future plant invasion studies.

## Methods

### Species and seed source

*Pascopyrum smithii*, and *B. inermis* are both rhizomatous, clonal, C_3_ grasses that begin flowering in late spring^60^. *Pascopyrum smithii* is native to North America and most abundant in northern mixed-grass prairie, which usually receives between 254 and 508 mm of precipitation annually. In its native range in China, *B. inermis* is known to occur in areas with around 400 mm of annual precipitation^61^. Both species are considered drought tolerant due to moderate to deep root systems and dehydration tolerance^62–64^. Although *B. inermis* maintains greater water-use efficiency than *P. smithii* at higher temperatures^65^, the production advantage of *B.inermis* over *P. smithii* disappears in seedlings but is maintained in adult plants when drought occurs^66, 67^. *Bromus inermis* was introduced to North America from Eurasia in the late 1880s for forage but has expanded outside its plantings and has invaded native prairie, including mesic tallgrass prairie, semi-arid northern mixed-grass prairie, and fescue prairies^30, 68–71^.

*Bromus inermis* and *P. smithii* seeds were respectively obtained from Dakota’s Best Seed LLC (Platte, South Dakota, USA) and Golden Willow Seeds, Inc. (Midland, South Dakota, USA). Seeds were germinated in Miracle-Gro® potting mix soil with a temperature regime of 16 °C night/22 °C day in a greenhouse at the South Dakota State University Seed Testing Laboratory in 2016. Because of their slower germination, *P. smithii* seeds were sown five days earlier than *B. inermis* to obtain the same growth stage at time of transplant. One single-leaf seedling was transplanted into each pot (16.5 cm dia. X 16.5 cm depth) filled with 600 g of non-fertilizer potting-soil (PRO-MIX® BX) and watered. A total of 480 pots were established (240 per species). Pots were watered to container capacity [(44–45% volumetric water content (VWC)); Decagon Devices, Soil Moisture Sensor: Model EC-5 calibrated to the potting soil] and seedlings acclimated to their pots for 18 days. One week after seedling transplant, each pot received 100 ml solution (0.343 g/L of Miracle-Gro® NPK (15-30-15); 5.8% ammoniacal nitrogen, 9.2% urea nitrogen; 30% P_2_O_5_; and 15% K_2_O). All methods were performed in accordance with relevant guidelines and regulations.

### Precipitation frequency treatment

Pots were watered every 2, 8, or 16 d to simulate high, medium, and low precipitation frequencies, respectively. Precipitation frequency treatments began 19 days after seedling transplant and were subsequently applied for 20 weeks. Each treatment developed predictable VWC patterns during the study (Fig. S1). Total water received was consistent across treatments and simulated average growing season precipitation of 51 mm mo^-1^ (based on 1981–2010 March–August precipitation of Rapid City Regional Airport, South Dakota; https://climate.sdstate.edu). Therefore, 2, 8, or 16 d treatments received 72 ml, 288 ml, and 576 ml of water during each watering event, respectively.

### Clipping treatment

A one-time clipping treatment was randomly assigned to half of the pots for each precipitation frequency treatment and species. This treatment was applied when seedlings of each species reached the three-collar leaf stage^72^, which is the typical grass development stage when livestock grazing is allowed on pastures by regional managers. Seedlings reached this stage 20 days after seedling transplant. Seedlings were clipped to a 4 cm stubble height to simulate early-season grazing by ungulates.

### Greenhouse conditions

Each treatment combination (species x precipitation frequency x clipping) was applied to 40 pots. Due to limited space, pots were randomly divided between two adjacent rooms of the greenhouse so that each treatment combination (species x clipping x precipitation frequency) was evenly represented in each room (*n* = 20). After seedlings acclimated to their pots for 18 days, precipitation frequency and clipping treatments were applied and plants were grown for 20 additional weeks. The photoperiod and temperature regime mimicked mixed-grass prairie field conditions during the growing season (June–October; Table S7). As the 20 week treatment period of the experiment began July 1, supplemental light (six 400 W high pressure sodium lamps) extended the ambient daylength between 0.5 and 1.5 h depending on the month (Table S7). Due to a greenhouse roof leak altering water availability in 42 pots in one room, each treatment combination in that room had between 10 and 20 replicates when these pots were removed from the experiment.

### Bud, rhizome, and tiller classification

After 20 weeks at which time plants would be entering the winter dormant season, plants were destructively harvested and washed free of soil. All rhizomes and adult tillers were counted. Rhizomes were belowground stems at least 0.5 cm in length with elongated internodes. Adult tillers had vertically elongated aboveground past their bud’s prophyll and had reached 3.6 cm and 4.5 cm in height for *B. inermis* and *P. smithii* respectively^42^. Three generations of tillers occurred during the experiment. Therefore, tillers and rhizomes were classified by generation^44^ (Fig. 6).

Three tillers and three rhizomes from each generation per plant were randomly selected to assess bud production and bud development stages. Each was examined under 6.7× to 45× magnification (Olympus® Stereo Microscope). Belowground buds, juvenile rhizomes, and juvenile tillers were counted, assessed to be living or dead, and classified by their size^42^. Collectively, live buds, small juvenile tillers, and large juvenile tillers were called live propagules.

Vegetative reproduction was assessed through new tiller production and the belowground bud bank formation of these young plant individuals. Spatial expansion ability of these seedlings was assessed through their rhizome production and rhizome length.

### Statistical analysis

Linear mixed models were used to analyze the effect of clipping, precipitation frequency, and species on propagule development, live propagule supply, new tillers established per tiller of each generation, and overall plant establishment measured by total tillers, total rhizomes, and total rhizome length per plant (PROC GLIMMIX)^74^. The treatments of clipping, precipitation frequency, and species were applied to the experimental unit of pot (i.e., plant). The generation factor was applied to the experimental unit of tiller, and the experimental unit of developmental stage was applied to the portion of the pot containing each developmental stage. Kenward–Roger’s (KR) method was used to approximate the denominator degrees of freedom, but the containment method (CON) was used for total rhizomes per plant and number of live propagules per tiller by generation.

Both the number of total tillers per plant and the number of total rhizomes per plant were analyzed using a negative binomial distribution in a three-way factorial treatment structure with the factors of precipitation frequency, clipping, and species in a randomized complete block design, with greenhouse room as the block effect. Total rhizome length per plant was analyzed using a gamma distribution in a three-way factorial treatment structure with the factors of precipitation frequency, clipping, and species in a randomized complete block design, with greenhouse room as the block effect. Tiller replacement (i.e., the number of new tillers established per tiller in each generation) was analyzed using a negative binomial distribution in a four-way factorial treatment structure with the fixed factors of precipitation frequency, clipping, species, and generation (three levels) in a randomized, split-plot design, blocking by greenhouse room. Because treatment factors were applied at different levels of experimental units creating a split-plot design, precipitation frequency, clipping, and species were analyzed at the whole-plot of pot, while generation was analyzed at the sub-plot of tiller.

For each plant, live propagules per plant was calculated by summing the number of propagules belonging to each generation of adult tillers or rhizomes. These quantities for each stem type within a plant were obtained using the propagule count per stem type (e.g., primary rhizome, secondary tiller, etc.) multiplied by the total stems of that type. The total number of live propagules per plant was analyzed using a gamma distribution in a three-way factorial treatment structure with the fixed factors of precipitation frequency, clipping, and species in a randomized complete block design, with the greenhouse room as the block effect. The number of live propagules per plant according to developmental stage was analyzed using a gamma distribution in a four-way factorial treatment structure with the fixed factors of precipitation frequency, clipping, species, and development stage in a randomized, split-plot design, blocking by greenhouse room. Because treatment factors were applied at different levels of experimental units creating a split-plot design, precipitation frequency, clipping, and species were analyzed at the whole-plot of pot, while developmental stage was analyzed at the sub-plot of a portion of the pot.

## Supplementary Information

Supplementary Information.

## Data Availability

The data that support the findings of this study are currently available from the corresponding author upon reasonable request.
