# Supplementary material for: Belowground mechanism reveals climate change impacts on invasive clonal plant establishment
Source: Sci Rep. 2022 Feb 21;12:2860. doi: 10.1038/s41598-022-06918-w (PMC8861118; doi:10.1038/s41598-022-06918-w)
Supplement: Supplementary file 1 — Supplementary Information. [file 41598_2022_6918_MOESM1_ESM.pdf]

Supplementary Information

Belowground mechanism reveals climate change impacts on invasive clonal plant establishment

Surendra Bam, Jacqueline P. Ott, Jack L. Butler, Lan Xu

Table S1. Total tillers per plant statistical model output.

Effect	Numerator DF	Denominator DF	F- Value	p-value
**Species**	1	9.026	195.59	<0.0001
**Frequency**	2	8.968	48.50	<0.0001
Clip	1	8.944	0.77	0.4031
**Species*Freq**	2	8.975	72.97	<0.0001
Species*Clip	1	8.944	1.42	0.2634
Freq*Clip	2	8.939	0.59	0.5724
Sp*Freq*Clip	2	8.939	1.74	0.2294

Notes: Frequency refers to precipitation frequency. Bolded effects are considered statistically significant.

Table S2. Total rhizomes per plant statistical model output.

Effect	Numerator DF	Denominator DF	F- Value	p-value
Species	1	11	4.64	0.0542
**Frequency**	2	11	31.23	<0.0001
**Clip**	1	11	7.86	0.0171
**Species*Freq**	2	11	19.13	0.0003
Species*Clip	1	11	0.67	0.4289
Freq*Clip	2	11	1.31	0.3092
Sp*Freq*Clip	2	11	3.34	0.0735

Notes: Frequency refers to precipitation frequency. Bolded effects are considered statistically significant.

Table S3. Total rhizome length per plant statistical model output.

Effect	Numerator DF	Denominator DF	F- Value	p-value
**Species**	1	9.026	195.59	<0.0001
**Frequency**	2	8.968	48.5	<0.0001
Clip	1	8.944	0.77	0.4031
**Species*Freq**	2	8.975	72.97	<0.0001
Species*Clip	1	8.944	1.42	0.2634
Freq*Clip	2	8.939	0.59	0.5724
Sp*Freq*Clip	2	8.939	1.74	0.2294

Notes: Frequency refers to precipitation frequency. Bolded effects are considered statistically significant.

Table S4. New tillers established per tiller statistical model output.

Effect	Numerator DF	Denominator DF	F- Value	p-value
**Species**	1	35.25	4.87	0.0339
**Frequency**	2	34.63	23.39	<0.0001
**Clip**	1	35.25	10.75	0.0023
**Generation**	2	34.34	1403.59	<0.0001
**Species*Freq**	2	34.63	18.61	<0.0001
Species*Clip	1	35.25	2.68	0.1103
Freq*Clip	2	34.63	2.94	0.0662
**Species*Gen**	2	34.34	52.17	<0.0001
**Freq*Gen**	4	33.74	16.83	<0.0001
Clip*Gen	2	34.34	1.75	0.1887
Sp*Freq*Clip	2	34.63	2.32	0.1133
**Sp*Freq*Gen**	4	33.74	14.58	<0.0001
**Sp*Clip*Gen**	2	34.34	4.35	0.0206
Freq*Clip*Gen	4	33.74	1.48	0.2312
Sp*Freq*Clip*Gen	4	33.74	1.01	0.4174

Notes: Frequency refers to precipitation frequency. Bolded effects are considered statistically significant.

Table S5. Live propagules per plant statistical model output.

Effect	Numerator DF	Denominator DF	F- Value	p-value
**Species**	1	8.421	19.19	0.0021
**Frequency**	2	8.404	15.43	0.0015
Clip	1	8.398	3.14	0.1123
**Species*Freq**	2	8.405	17.09	0.0011
Species*Clip	1	8.398	0.76	0.4065
Freq*Clip	2	8.394	2.05	0.188
Sp*Freq*Clip	2	8.394	0.14	0.8722

Notes: Frequency refers to precipitation frequency. Bolded effects are considered statistically significant.

Table S6. Proportion of live propagules per plant statistical model output.

Effect	Numerator DF	Denominator DF	F- Value	p-value
**Species**	1	33.78	15.12	0.0004
Frequency	2	33.41	0.84	0.439
Clip	1	33.24	1.12	0.2974
**DevStage**	2	33.24	951	<0.0001
Species*Freq	2	33.43	0.98	0.3855
**Species*Clip**	1	33.24	4.8	0.0355
Freq*Clip	2	33.26	1.6	0.2171
**Species*DevStage**	2	33.24	10.29	0.0003
Freq*DevStage	4	33.23	1.36	0.2694
Clip*DevStage	2	33.24	0.43	0.6513
Sp*Freq*Clip	2	33.28	0.34	0.712
**Sp*Freq*DevStage**	4	33.23	4.1	0.0083
Sp*Clip*DevStage	2	33.24	2.44	0.1026
Freq*Clip*DevStage	4	33.23	0.64	0.6405
Sp*Freq*Clip*DevStage	4	33.23	1.25	0.3106

Notes: Frequency refers to precipitation frequency. DevStage refers to developmental stage. Bolded effects are considered statistically significant.

Table S7. Day and night temperatures and photoperiod for greenhouse.

**Month[1]**	**Day Temp. (°C)**	**Night Temp. (°C)**	**Day Length[2] (hr)**	**Artificial Light (hr)**
June	19.5	16.0	15.5	0.5
July	23.6	19.5	15.0	1.0
August	21.5	17.7	14.0	1.5
September	17.6	13.3	12.5	1.5
October	9.5	6.5	11	1.5

1-Each simulated month was 4 weeks of the 20 week experiment. Values were based off of 10 years of climate data from Rapid City, South Dakota. 2- Total day length including artificial light used to extend ambient day length.

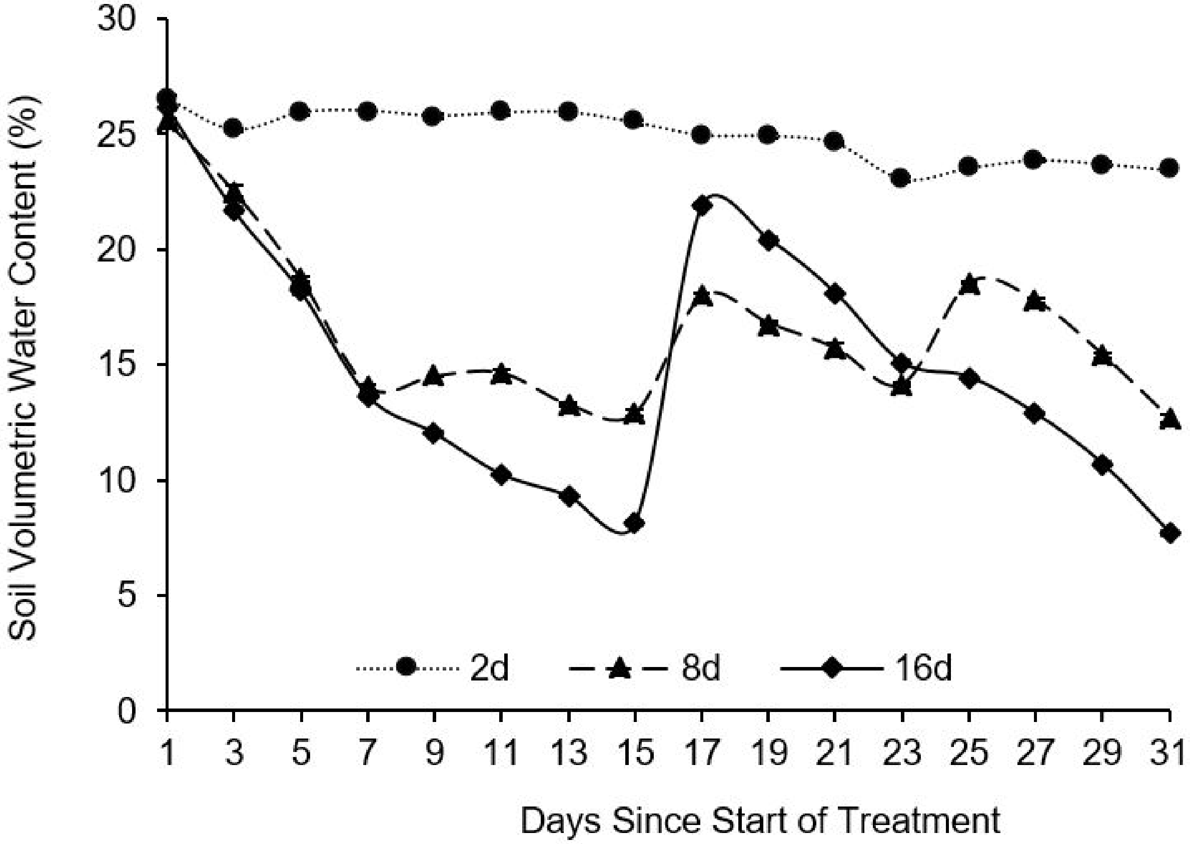

Fig. S1 Soil volumetric water content (VWC) of 2 day (2d), 8 day (8d), and 16 day (16d) precipitation frequency treatments. Each point represents mean ± SE (*n* = 20). VWC was measured every 2 days for the first 4.5 weeks (shown) and then once every 8 days for an additional 5.5 weeks (not shown) to ensure the oscillation patterns displayed during the first 4.5 weeks days persisted throughout the whole experiment. The average VWC for 2d, 8d and 16d during the subsequent 5.5 weeks (approximately week 4 to 12) of the experiment were 21.7% ± 0.15, 13.27 ± 0.22, and 8.89 ± 0.16, respectively (not shown).
